# Screening and characterization of PHA producing bacteria from sewage water identifying *Bacillus paranthracis* RSKS-3 for bioplastic production

**DOI:** 10.1186/s12866-025-03841-8

**Published:** 2025-03-14

**Authors:** Rohan Samir Kumar Sachan, Abhinav Kumar, Arun Karnwal, Prabhu Paramasivam, Ashish Agrawal, Abinet Gosaye Ayanie

**Affiliations:** 1 Department of Microbiology, School of Bioengineering and Biosciences, Lovely Professional University, Phagwara, Punjab 144411 India; 2 Department of Nuclear and Renewable Energy, Ural Federal University Named after the First President of Russia Boris Yeltsin, Ekaterinburg, 620002 Russia; 3 Department of Research and Innovation, Saveetha School of Engineering, SIMATS, Chennai, Tamil Nadu 602105 India; 4 Centre for Research Impact & Outcome, Chitkara University Institute of Engineering and Technology, Chitkara University, Rajpura, Punjab 140401 India; 5 Department of Mechanical Engineering, Adama Science and Technology University, Adama, 2552 Ethiopia; 6 Department of Technical Sciences, Western Caspian University, Baku, AZ1033 Azerbaijan; 7 Refrigeration &Air-condition Department, Technical Engineering College, The Islamic University, Najaf, Iraq; 8 Department of Medical Laboratory Sciences, School of Allied and Healthcare Sciences, GNA University, Phagwara-144401, Punjab, India; 9 Department of Microbiology, Graphic Era (Deemed to be University), Dehradun-248009, Uttarakhand, India

**Keywords:** Polyhydroxyalkonate, Biocompatible, Biodegradable, Bioplastic, Sudan Black B, Sewage, Polyethylene, Renewable source

## Abstract

Polyhydroxyalkanoate (PHA) as bioplastic is considered a replacement for conventional plastic due to its more beneficial properties. The ability of PHA to biodegrade in a shorter period is a major advantage. Different sewage water samples were collected from the Budha Nala near the Maheru regions of Punjab. PHA-producing bacteria were isolated using minimal salt media supplemented with Nile blue. Further screening was carried out using Sudan Black B stain and Nile red stain. The positive isolates were characterized for gram reaction, motility, and biochemical tests. The individual isolates were later screened for maximum PHA accumulation using minimal salt supplemented with glucose. The extracted PHA was characterized using FTIR, XRD, SEM, UV spectroscopy, NMR, and TGA. Twenty-six different PHA-producing bacteria were isolated on minimal salt media supplemented with Nile blue. Upon Sudan Black B stain and Nile red stain, nineteen isolates showed black granules and orange fluorescence bodies under 100X magnification that confirmed polyhydroxyalkanoates. The biochemical tests partially characterized isolates belonging to the *Bacillus* genus. All the isolates produced PHA in granular form, however, isolate P-3 showed maximum production of 0.068 g/L. The extracted PHA was characterized using FTIR and XRD for its chemical and crystallinity studies and the UV spectroscopy confirmed the extracted PHA by analyzing absorption spectra at 235 nm of standard crotonic acid and sulfuric acid conversion of PHA to crotonic acid. The isolated P-3, *Bacillus paranthracis* RSKS-3 is the first reported bacterium to produce polyhydroxyalkanoates. Further studies is necessary to optimize the production efficiency of the bacterium for maximum PHA yield.

## Introduction

Plastic is a synthetic material made from polymers, which are long chains of molecules. It is one of the most used materials worldwide due to its durability, versatility, and low cost of production [1]. Plastics are used in a wide range of applications, including packaging, construction, electronics, automotive parts, and medical devices [2, 3]. Plastic waste generation has become a significant global environmental issue. Only 9% of the plastic waste is recycled and over 22% is mishandled [4]. The durability and resistance to degradation that make plastic useful also contribute to its persistence in the environment. Plastic waste can take hundreds of years to decompose naturally, causing serious pollution problems. The production and consumption of plastic have increased exponentially over the past few decades. For instance, in 2023, global plastic production surged to 413.8 million metric tons, a significant rise from 1.5 million metric tons recorded in 1950 [5]. The World Economic Forum estimated that global plastic production reached over 420 million metric tons in 2019 [6]. Unfortunately, a significant portion of this plastic ends up as waste. Improper plastic waste management, including inadequate recycling infrastructure and improper disposal, has led to the accumulation of plastic in landfills, oceans, and other ecosystems. Plastic pollution poses a threat to wildlife, as animals can mistake it for food or become entangled in it. Furthermore, plastics can break down into microplastics, which are tiny particles that can contaminate water sources, soil, and even the air we breathe [7, 8]. Efforts to address the plastic waste problem are being made worldwide. Many countries have implemented measures to reduce single-use plastics, promote recycling, and encourage the development of more sustainable alternatives. Additionally, awareness campaigns and initiatives aimed at educating the public about the environmental impact of plastic waste are increasing [9]. Petroleum-based plastics possess a threat to the ecosystem and the main cause is their non-degradable nature. This has created chaos in solid waste management due to piling up of plastic wastes across the globe [10]. However, its immense popularity from industrial to household usage has led to extensive usage.

In recent years, biopolymers have emerged as a promising alternative to traditional plastics to address the environmental concerns associated with plastic waste [11]. Biopolymers are polymers derived from renewable sources such as plants, bacteria, or algae, and they offer several advantages over conventional plastics [12]. One of the main advantages of biopolymers is their biodegradability. Unlike traditional plastics, which can persist in the environment for hundreds of years, biopolymers can break down naturally through biological processes (microbial degradation, microbial photodegradation, etc.) [13, 14], reducing the long-term environmental impact. Biodegradable biopolymers can be composted or degraded by microorganisms, leading to a more sustainable waste management system [15]. For instance, microbial degradation of PHA involves enzymatic hydrolysis by PHA depolymerases, breaking it into monomers, which are then assimilated into microbial metabolism and mineralized into CO_2_, water, and biomass [16, 17]. Additionally, the production of biopolymers typically requires less energy and generates fewer greenhouse gas emissions compared to traditional plastics derived from fossil fuels [18]. Biopolymers derived from plant-based sources, such as corn or sugarcane, can help reduce our reliance on finite resources and contribute to a more circular economy. Biopolymers also offer versatility in terms of their properties and applications. They can be tailored to meet specific requirements, making them suitable for a wide range of products, including packaging materials, disposable cutlery, and even medical devices [19]. Research and development in the field of biopolymers are ongoing, with efforts focused on improving their mechanical strength, barrier properties, and heat resistance to expand their potential applications [20]. However, it's important to note that while biopolymers offer environmental advantages, they are not a complete solution to the plastic waste problem. Proper waste management systems and infrastructure for the collection and composting of biopolymers are crucial to ensure their sustainable disposal.

PHA offers distinct advantages over other biopolymers like PLA and starch-based plastics due to its natural microbial production and complete biodegradability in diverse environments, including soil, marine, and compost settings. Unlike PLA, which requires industrial composting, PHA undergoes enzymatic degradation, making it more sustainable. It also exhibits superior thermal stability and mechanical properties, closely resembling conventional plastics. Additionally, PHA is biocompatible and non-toxic, making it ideal for medical and packaging applications [21, 22]. In the pursuit of advancing our understanding of polyhydroxyalkanoate (PHA) biosynthesis, this research unfolds through a methodical sequence of investigative stages. Commencing with the isolation of PHA-producing bacteria derived from sewage samples in the Maheru village, a rigorous screening protocol is applied to discern strains manifesting significant PHA production capabilities. Following the identification of PHA-producing bacteria, a sophisticated genomic characterization ensues, with a specific emphasis on the detailed profiling of PHA-related genes. This genetic scrutiny aims to illuminate the molecular intricacies inherent in PHA biosynthesis within the selected strains. Transitioning from genomic exploration to practical application, the study proceeds to cultivate the chosen strains for PHA synthesis. The final phase encompasses an exhaustive characterization of the produced PHA, employing advanced biophysical techniques to scrutinize fundamental properties, including crystallinity, molecular weight distribution, and structural composition. This integrated approach, spanning bacterial isolation, screening, genomic characterization, PHA production, and polymer analysis, aspires to contribute substantively to the academic discourse surrounding PHA biosynthesis, with potential implications for the development of sustainable and biodegradable materials.

## Materials and methods

### Sample procurement and identification of polyhydroxyalkanoate-producing bacteria

Four sewage samples were collected from areas of Budha Nala, Punjab, India. The locations are Location 1: 31.2520761 Latitude, 75.6812032 Longitude; Location 2: 31.2704923 Latitude, 75.6903445 Longitude; Location 3: 31.2533141 Latitude, 75.6639076 Longitude; Location 4: 31.2268515 Latitude, 75.6341338 Longitude. Approximately, 10 mL of sewage water was drawn in 50 mL sample bottles and transported to the School of Bioengineering and Biosciences, Lovely Professional University. The samples were refrigerated at 4 ℃ up to 2 weeks for further analysis.

One mL of each sample was inoculated in 10 mL sterile nutrient broth and labeled as mother cultures. The samples were then serially diluted and plated on Nile Blue minimal salt media having media composition: sucrose (20 g/L); ammonium chloride (0.35 g/L); magnesium sulfate (0.2 g/L); potassium dihydrogen phosphate (2.65 g/L); calcium chloride (0.05 g/L); ferric chloride (0.01 g/L); zinc sulfate (0.01 g/L); Nile blue (0.225 mg/L); agar (20 g/L), and pH was adjusted to 7.0. The plates were incubated at 37 ℃ for 72 h. The colonies obtained were illuminated under UV light for preliminary screening. The white luminescence from the colonies was considered potential PHA-producing colonies and was sub-cultured for primary and secondary screening.

### Screening of PHA-producing isolates

For the primary screening of PHA bodies, an alcoholic solution of Sudan Black B stain was used [23]. The smear was prepared on a glass slide and heat-fixed. The smear was flooded with 0.3% alcoholic Sudan Black B (0.3 g in 100 mL 95% ethanol) stain for 15–20 min. Later, the excess stain was drained off and was counterstained with safranin for 5 min. The stain was washed off with tap water, air-dried, and observed under 100X microscopy.

For secondary screening, Nile red agar plates were prepared using composition: sucrose (20 g/L); ammonium chloride (0.35 g/L); magnesium sulfate (0.2 g/L); potassium dihydrogen phosphate (2.65 g/L); calcium chloride (0.05 g/L); ferric chloride (0.01 g/L); zinc sulfate (0.01 g/L); Nile red (0.5 μg /mL); agar (20 g/L), and pH was adjusted to 7.0 [24]. The isolates were streaked on the agar plates and incubated for 72 h at 37 ℃. After the incubation, the plates were UV-illuminated for the presence of bright orange fluorescence for PHA bodies.

### Morphological and biochemical characterization of PHA-producing bacteria

Characterization of the morphology and biochemistry of bacteria that were found to be capable of producing polyhydroxyalkanoates was done. To differentiate between Gram-positive and Gram-negative classifications, Gram reaction tests were also used. Different biochemical investigations have been conducted on PHA-producing bacteria, such as the indole synthesis test, methyl-red test, Voges–Proskauer reaction, citrate utilization, urease activity, oxidase and catalase activity, H_2_S test, nitrate reduction, and triple iron sugar test. Also, growth characteristics were analyzed on mannitol salt agar and spirit blue agar. Furthermore, an array of carbohydrates was investigated, namely glucose, maltose, fructose, galactose, lactose, and mannitol [13, 25, 26].

### Molecular characterization

#### DNA isolation: qualitative and quantitative analysis

Using the Qiagen DNeasy UltraClean Microbial Kit (Cat. No. 12224–50), DNA was isolated from the provided sample. A 0.8% agarose gel was used to assess the genomic DNA's quality following DNA isolation. DNA fragments can be separated according to size using an apparatus called agarose gel electrophoresis. After loading a small volume (5 μl) of the isolated DNA sample onto the gel, it was run for 30 min at 110 V. A single intact band on the gel is indicative of high-quality genomic DNA [27, 28]. The sample was loaded into the BioTeK Epoch spectrophotometer with 2 μl to measure the DNA purity (A260/280 ratio) of the genomic DNA.

#### Genome sequencing and its assembly

Thermo Fisher Scientific, USA's Ion XpressTM Plus Fragment Library Kit was used to prepare the libraries in accordance with manufacturing instructions, which included steps for fragmentation, purification of fragments, ligation, amplification, and quantification. The Ion Library Taqman Quantitation kit was utilised for the quantitation step. In the purified fragmented DNA step, the fragment size was examined (QC Step). This was carried out using the AgilentTM 2100 Bioanalyzer and the AgilentTM High Sensitivity DNA Kit by the instructions [29].

Following the library preparation, the Ion OneTouchTM 2 System was used to prepare the template (Template Preparation Step) in accordance with the manufacturer's instructions for the Ion 540TM Kit (Thermo Scientific, USA). After that, the library was sequenced using the Ion GeneStudio S5 Plus System (Ion Torrent, Thermo Scientific, USA) and loaded onto a chip using the Ion 540TM Chip Kit. The genome was sequenced and assembled using SPAdes v3.13.0 and the assessment of genome was done using CheckM v1.2.1.

#### Gene analysis and its annotation

Reads from Ion Torrent single-end sequencing was put through quality trimming and adapter using Trim Galore and Cutadapt, with a 20 quality phred score cutoff. With SPAdes v3.13.0, the high-quality reads that were obtained were assembled from scratch [30]. The contig length (> 1000 bp) of the resulting contigs was used to filter them. QUAST was used to generate the assembly statistics. CheckM v1.2.1 was used to evaluate the assembled genomes for contamination and completion.

The NCBI Prokaryotic Genome Annotation Pipeline (PGAP) algorithm and the BV-BRC (PATRIC) server were used to annotate the final assembled genomes. By using BlastKOALA to search the Kyoto Encyclopaedia of Genes and Genomes (KEGG) database, the cellular functions of the proteins encoded in the genome and their functional classification were deduced. Kegg Orthology (KO) was used to determine metabolic features and pathways. Kegg Mapper was used to reconstruct the metabolic pathway using the KO number that BlastKOALA assigned to each protein sequence in the genome. By uploading classified gene symbols into the PANTHER Classification System, GO annotation was obtained for annotated proteins. A circular genome was created using MGCplotter, and COGclassifier was used to classify predicted genes into COG (Cluster of Orthologous Genes) functional categories.

#### Phylogenetic study analysis

Accurate intergenomic distances were inferred using the distance formula d5 and the algorithm 'trimming' for the phylogenomic inference. All pairwise comparisons within the set of genomes were carried out using GBDP. A total of 100 distance replicates were computed. GGDC 4.0 recommended settings were used to calculate digital DDH values and confidence intervals.

To find comparable public genomes or calculate genome distance estimation using Mash, the BV-BRC (PATRIC) server's Similar Genome Finder Service was utilized. A set of genomes that meet the given similarity criteria are returned. The Mash (v2.3) programme was manually run to confirm the results.

A thorough genome analysis was used to build the general phylogenetic tree using the codon tree method from single-copy genes using the programme RAxML, and 77 close reference genomes were chosen using the BV-BRC similar genome finder services.

#### Sequence accession number

The whole genomic sequence of *Bacillus paranthracis* RSKS-3 has been registered in GenBank with the bio-sample accession number SAMN39897631.

#### PHA gene identification in *B*. *paranthracis* RSKS-3

For the identification of *pha* genes in *B*. *paranthracis* RSKS-3, primers were designed and synthesized [31]. Sequences of primers are as follows in Table 1.

**Table 1:** Primer design for the identification of *pha* genes in *Bacillus paranthracis* RSKS-3

Primer type	Primer sequence	PHA genes	PHA specific restriction enzyme
Forward	CGCCATATGATGATTGATCAAAAATTC	*phaR*	NdeI
Reverse	CCGGAATTCTCATTTTTTATTTTCTGGCTTATTC		EcoRI
Forward	CGCCATATGATGGTTCAATTAAATGGAAAAG	*phaB*	NdeI
Reverse	CCGGAATTCTTACATATATAATCCGCCGTTAATG		EcoRI
Forward	CGCGGATCCATGACTACATTCGTAACAGAATG	*phaC*	BamHI
Reverse	CCGCTCGAGTTACTTCGAACGCTCGTCAAG		XhoI

The specificity and efficiency of these primer sets were paramount, ensuring selective amplification of the desired genomic regions associated with the *pha* genes. Subsequently, PCR reactions for each *pha* genes (*phaR*, *phaB*, and *phaC*) were established, delineating the specifics of the reaction conditions as outlined in Table 2, 3, and 4. This comprehensive approach aimed to standardize and optimize the PCR conditions across the different *pha* genes, maintaining consistency and reliability in the experimental setup.

**Table 2:** Thermal profiling (PCR conditions) of *PhaR*

Steps	Temperature	Time	Cycles
Initial denaturation	94 °C	1 min	X1
Final denaturation	98 °C	10 s	X32
Annealing	55 °C	15 s	
Initial extension	68 °C	15 s sec	
Final extension	68 °C	3 min	X1
Hold	12 °C	∞	X1

**Table 3:** Thermal profiling (PCR conditions) of *PhaB*

Steps	Temperature	Time	Cycles
Initial denaturation	94 °C	1 min	X1
Final denaturation	98 °C	10 s	X32
Annealing	58 °C	15 s	
Initial extension	68 °C	30 s	
Final extension	68 °C	3 min	X1
Hold	12 °C	∞	X1

**Table 4:** Thermal profiling (PCR conditions) of *PhaC*

Steps	Temperature	Time	Cycles
Initial denaturation	94 °C	1 min	X1
Final denaturation	98 °C	10 s	X32
Annealing	64 °C	15 s	
Initial extension	68 °C	1:30 min	
Final extension	68 °C	3 min	X1
Hold	12 °C	∞	X1

However, SnapGene, a comprehensive molecular biology software, played a pivotal role in proving the theoretical gene amplification of *pha* genes R, B, and C [32]. The experimental process involved in-silico PCR of *pha* genes using specific primers using SnapGene's PCR design tools. The primers were employed in conjunction with chosen restriction enzymes (Table 1) to amplify the *pha* genes. Restriction enzymes, selected based on the known DNA sequences of the *pha* genes, played a key role in preparing the DNA samples for PCR by cutting them into fragments suitable for amplification. This strategic combination of specific primers and restriction enzymes provided a targeted and controlled approach, ensuring the selective amplification of *pha* genes R, B, and C.

### Submerged fermentation and recovery of PHA production

The isolates were screened through primary, secondary, gram staining, and biochemical analysis and were assessed to produce PHA. The cultures were grown in sterile nutrient broth and later adjusted to 10^8^ cells/ mL using sterile distilled water. For the production, 50 mL media (glucose (10 g/L), yeast extract (2.5 g/L), magnesium chloride (0.2 g/L), sodium chloride (0.5 g/L), and peptone (2.5 g/L) was drawn into 100 mL Erlenmeyer flasks, and 1 mL of the culture was inoculated and incubated at 37 ℃ for 72 h in a rotatory incubator at 150 RPM [33].

The liquid component underwent centrifugation at 8000 RPM for 10 min to gather the cell biomass. Cellular biomass was solubilized by adding 1 ml of distilled water to preweighed Eppendorf tubes. After a second round of centrifugation at 10,000 RPM, the liquid part was eliminated from the tubes. Subsequently, the solid residue was dried at 60 °C until it reached a constant weight. The pellets were treated with a 6% sodium hypochlorite (NaClO) solution to remove cellular debris [4, 33, 34]. Later, placed in an orbital shaker incubator at 30 °C for two hours. After incubation, the tubes were centrifuged at 8000 RPM for twenty minutes. The solid particles were washed with distilled water after the liquid component was separated. The liquid was moved to Eppendorf tubes and centrifuged at 10,000 g for twenty minutes. The liquid portion above the sediment was then dumped. The residual cellular waste was eliminated by rinsing the solid mass with acetone. The pellets were dried at 60 °C to maintain a consistent weight.

### Characterization of PHA

The characterization of PHA produced was done with biophysical techniques.

#### Fourier transformed infra-red microscopy

Using Perkin-Elmer equipment of the US, we used ATR-FTIR (Attenuated Total Reflectance Fourier Transform Infrared Spectroscopy) to examine the functional groups that were present in the extracted and control PHA samples. The wavelength range examined in the analysis was 4000–400 cm^−1^ [4].

#### X-ray diffraction

XRD spectroscopy was used to examine the crystalline structure of the obtained Polyhydroxyalkanoate (PHA) particles. The device used a radiation wavelength (k) of 1.5405 Å to scan a range between 1 and 70˚ within 2θ [35].

#### Scanning electron microscopy

The microstructure, surface morphology, and elemental composition of the extracted polyhydroxybutyrate (PHB) were analyzed using a scanning electron microscope (SEM) equipped with Energy-dispersive X-ray spectroscopy (EDS). The SEM used for this study was a Jeol JSM-7600F equipment, operating at a voltage of 20 kV.

#### UV spectroscopy

One mg of dried PHA granules were dissolved in 2 mL of concentrated sulfuric acid and was brought to a boiled for 10 min. Then, using a calibration baseline of sulfuric acid and standard crotonic acid from the Central Drug House Laboratory Reagent in New Delhi, India, UV spectra were recorded between 800 and 200 nm.

#### Nuclear resonance microscopy

A 5-mm 1H-probe fitted to a BRUAK AV-400 spectrometer and same for 13C was used to acquire the NMR spectra of 1-mL sample of Polyhydroxyalkanoates. The solvent used was deuterated chloroform (CDCl_3_) at a concentration of 10 g/L. PHA's 1H-NMR and 13C spectra was recorded at 400 MHz.

#### Thermo-Gravimetric analysis

Using a Mettler TG50 thermobalance, the thermal stability of the samples was evaluated. On the instrument, 5 mg of dried samples were measured, and they were examined at a nitrogen flow rate of 20 mL per minute. The temperature was raised to 500 °C at a rate of 10 °C per minute.

## Result and discussion

### Sample collection and analysis

#### Isolation and screening of PHA-producing bacteria

**Fig. 1:**
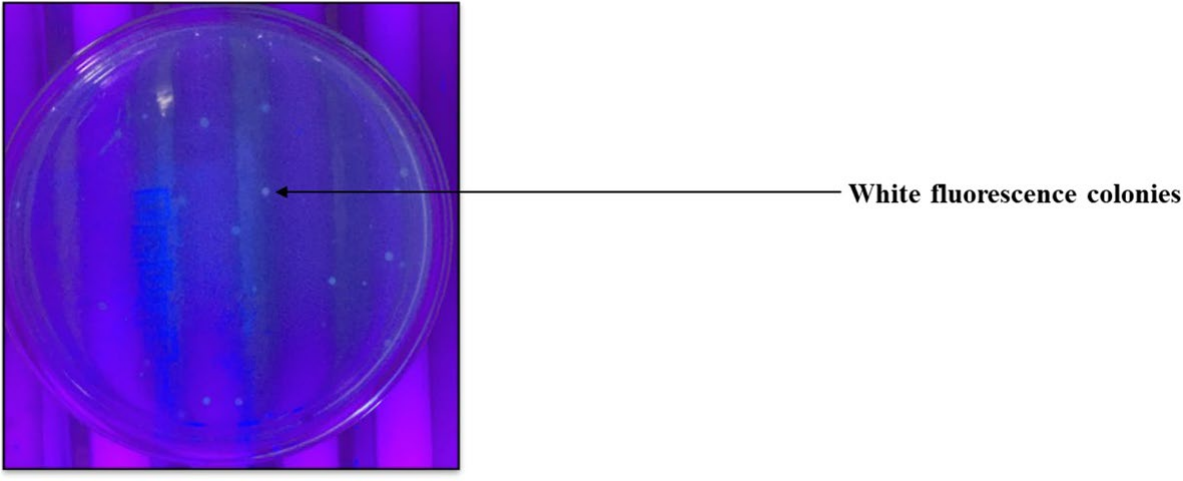
UV-exposed white fluorescence colonies preliminary screen potential PHA producers

**Fig. 2:**
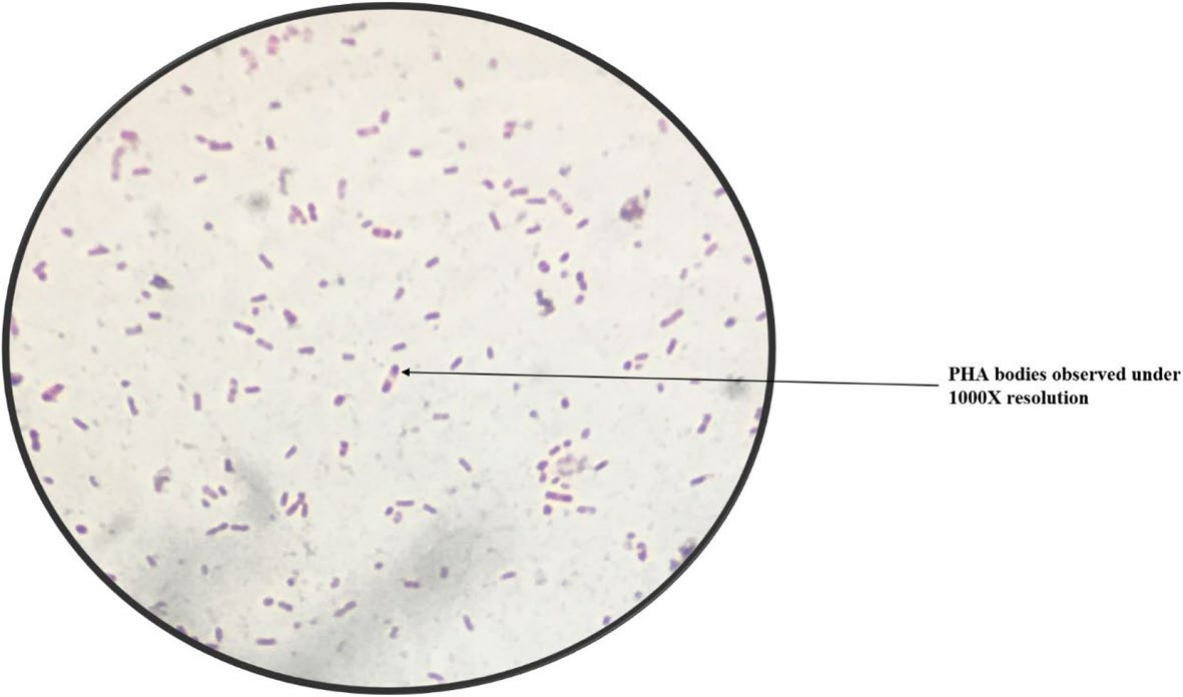
Sudan Black B stain revealing black-colored bodies (indicative of PHA) under 100X magnification

**Fig. 3:**
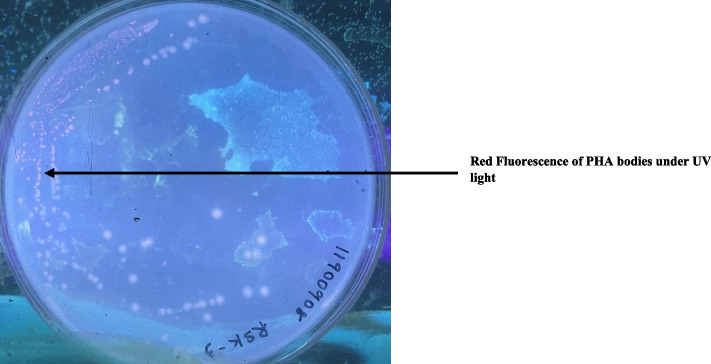
Nile red stain revealing orange fluorescence bodies (indicative of PHA) under UV illumination

A total of 59 isolates were recovered from four sewage bodies of the Maheru village. Of these, 26 showed white fluorescence under UV light (Fig. 1), thus suggesting preliminary they could produce PHAs in their cells. Microscopic analysis of Sudan Black B showed the presence of black granules in all 26 isolates (Fig. 2,Table 5). All the isolates showed orange fluorescence upon exposure to UV light after growth in media containing Nile Red dye (Fig. 3,Table 6).

**Table 5:** Observations of Sudan Black B

Isolates no	Observation
P-1	Positive
P-2	Positive
P-3	Positive
P-4	Positive
P-5	Positive
P-6	Negative
P-7	Positive
P-8	Negative
P-9	Positive
P-10	Negative
P-11	Positive
P-12	Positive
P-13	Positive
P-14	Positive
P-15	Negative
P-16	Positive
P-17	Negative
P-18	Positive
P-19	Positive
P-20	Positive
P-21	Positive
P-22	Negative
P-23	Negative
P-24	Positive
P-25	Positive
P-26	Positive

**Table 6:** Secondary screening of PHA producers

Isolates no	Observation
P-1	Orange fluorescence colonies
P-2	Orange fluorescence colonies
P-3	Orange fluorescence colonies
P-4	Orange fluorescence colonies
P-5	Orange fluorescence colonies
P-7	Orange fluorescence colonies
P-9	Orange fluorescence colonies
P-11	Orange fluorescence colonies
P-12	Orange fluorescence colonies
P-13	Orange fluorescence colonies
P-14	Orange fluorescence colonies
P-16	Orange fluorescence colonies
P-18	Orange fluorescence colonies
P-19	Orange fluorescence colonies
P-20	Orange fluorescence colonies
P-21	Orange fluorescence colonies
P-24	Orange fluorescence colonies
P-25	Orange fluorescence colonies
P-26	Orange fluorescence colonies

Bhuwal et al. [36] isolated 42 strains from pulp, paper, and cardboard industry wastewater, capable of accumulating PHA when stained with Sudan Black B [37]. Li et al. [38] isolated a bacterial strain from wastewater residual sludge upon treatment with propylene oxide saponification [38]. Mohapatra et al. [39] isolated 18 strains of PHA accumulating bacterial species from the Taladanda canal, Odisha, India [39]. All 18 strains showed positive black-blue granules when stained with Sudan Black B. Reddy et al. (2017) isolated 14 colonies from the outskirts of Guntur, Andhra Pradesh; among which 5 colonies were PHA-producing strains that showed positive black-blue granules when stained with Sudan Black B [40]

### Partial identification of PHA accumulation species

The partial identification of the PHA accumulation species was done according to Bergey’s manual of systematic bacteriology.

Gram reaction was done for morphological identification. The positive Sudan Black B isolates were stained with a gram reagent. Gram staining results revealed that many of the isolates from sewage water were gram-positive bacteria. The morphology of most gram-positive isolates were small rods. The observations for gram reactions are depicted in Table 7. Beyond the gram reaction, motility also adds another layer to the characterization. Through the hanging drop method, all the isolates were motile under a 40X bright field microscope (Table 8).

**Table 7:** Gram staining reaction

Isolates no	Observation
P-1	Gram-positive rods
P-2	Gram-positive rods
P-3	Gram-positive rods
P-4	Gram-positive rods
P-5	Gram-positive rods
P-7	Gram-positive rods
P-9	Gram-positive rods
P-11	Gram-positive rods
P-12	Gram-positive rods
P-13	Gram-positive rods
P-14	Gram-positive rods
P-16	Gram-positive rods
P-18	Gram-positive rods
P-19	Gram-positive rods
P-20	Gram-positive rods
P-21	Gram-positive rods
P-24	Gram-positive rods
P-25	Gram-positive rods
P-26	Gram-positive rods

**Table 8:** Motility test

Isolates no	Observation
P-1	Motile
P-2	Motile
P-3	Motile
P-4	Motile
P-5	Motile
P-7	Motile
P-9	Motile
P-11	Motile
P-12	Motile
P-13	Motile
P-14	Motile
P-16	Motile
P-18	Motile
P-19	Motile
P-20	Motile
P-21	Motile
P-24	Motile
P-25	Motile
P-26	Motile

The isolates had diverse biochemical features due to their ability to utilize various substrates, differentially. For instance, in carbohydrate catabolism, many isolates were found to metabolize glucose, fructose, mannitol, and lactose. Whereas maltose and galactose were not readily metabolized by some isolates. The biochemical tests of the isolates are shown in Table 9, 10.

**Table 9:** Biochemical tests of PHA producing bacterial isolates

NAME OF THE BIOCHEMICAL TESTS	ISOLATES OBSERVATIONS																		
	**P-1**	**P-2**	**P-3**	**P-4**	**P-5**	**P-7**	**P-9**	**P-11**	**P-12**	**P-13**	**P-14**	**P-16**	**P-18**	**P-19**	**P-20**	**P-21**	**P-24**	**P-25**	**P-26**
**Indole test**	+	+	+	+	-	+	+	+	+	+	+	+	+	+	+	+	+	+	+
**Methyl-red test**	-	+	-	-	-	-	+	-	-	+	-	-	-	+	-	-	-	+	-
**Voges-Proskauer test**	-	-	-	-	-	-	-	-	-	-	-	-	-	-	-	-	-	-	-
**Citrate utilization test**	+	+	+	+	+	+	-	+	-	+	+	-	+	+	-	-	+	+	+
**Starch hydrolysis test**	-	-	-	-	-	-	-	-	-	-	-	-	-	-	-	-	-	-	-
**Oxidase test**	-	+	+	-	+	+	+	-	+	+	+	+	+	+	+	-	+	+	+
**Catalase test**	+	+	+	+	+	+	+	+	+	+	+	+	+	+	+	+	+	+	+
**Urease test**	-	-	-	-	-	-	-	-	-	-	-	-	-	-	-	-	-	-	-
**Sulfide reduction test**	-	-	-	+	-	+	+	+	+	-	-	+	-	-	-	-	-	+	+
**Nitrate reduction test**	-	-	-	-	+	+	-	-	-	+	+	+	-	-	-	+	-	-	-
**Triple Sugar Iron test**	a/a*	a/a*	a/a*	a/a*	a/a*	a/a*	a/a*	a/a*	a/a	a/a	a/a*	a/a	a/a*	a/a	a/a*	a/a	a/a*	a/a*	a/a*
**Mannitol salt agar growth**	-	-	-	-	-	-	-	-	-	-	-	-	-	-	-	-	-	-	-
**Spirit blue agar growth**	+	-	+	+	+	+	+	-	-	-	-	-	+	-	+	+	-	-	-

‘ + ’ indicates positive result for the test

‘-’ indicates negative result for the test

‘a/a’ indicates acidic slant and butt

‘a/a*’ indicates acidic slant and but with gas production

‘NA’ indicates Not applicable

**Table 10:** Sugar fermentation by the isolates

Isolates	Sugar Utilization					
	**Glucose**	**Maltose**	**Fructose**	**Mannitol**	**Lactose**	**Galactose**
**P-1**	a*	a*	a*	a*	a*	a*
**P-2**	a*	a*	a*	a*	a*	a*
**P-3**	a*	-*	a*	a*	**-**	a*
**P-4**	a*	a*	a*	a*	**-**	a*
**P-5**	a*	a*	a*	a*	**-**	a*
**P-7**	a*	-*	a*	**-**	a*	a*
**P-9**	a*	a*	a*	a*	a*	a*
**P-11**	a*	-*	a*	**-**	a*	a*
**P-12**	a*	-*	a*	a*	a*	a*
**P-13**	a*	-*	a*	a*	a*	a*
**P-14**	a*	-*	a*	a*	a*	a*
**P-16**	a*	-*	a*	a*	a*	-*
**P-18**	a*	-*	a*	a*	a*	a*
**P-19**	a*	-*	a*	a*	a*	a*
**P-20**	a*	-*	a*	a*	a*	-*
**P-21**	a*	-*	a*	a*	a*	a*
**P-24**	a*	+ *	a*	a*	a*	a*
**P-25**	a*	+ *	a*	a*	a*	a*
**P-26**	a*	+ *	a*	a*	a*	a*

### Molecular identification

The evolutionary history was inferred using the UPGMA method (Sneath and Sokal, 1973; Felsentein, 1985). The optimal tree is shown. The tree is drawn to scale, with branch lengths in the same units as those of the evolutionary distances used to infer the phylogenetic tree. The evolutionary distances were computed using the Kimura 2-parameter method (Kimura, 1980) and are in the units of the number of base substitutions per site. This analysis involved 16 nucleotide sequences. Codon positions included were 1st + 2nd + 3rd + Noncoding. All positions containing gaps and missing data were eliminated (complete deletion option). There were a total of 1218 positions in the final dataset. Evolutionary analyses were conducted in MEGA11 (Tamura and Kumar, 2021).

#### Gene annotation and assembly

The whole genome studies of *Bacillus paranthracis* have provided comprehensive insights into its genetic makeup, highlighting key features such as genome length, gene count, GC content, and various functional annotations. Bacillus paranthracis RSKS-3 revealed a genome length of 5,130,571 base pairs with a total of 5401 genes and a GC content of 35.38%. Similar studies by Baev et al. [41] on *Bacillus paranthracis* PUMB_17 revealed a genome length of 5,295,234 base pairs (bp) with a total of 5,487 genes and a GC content of 35.2% [41]. Similarly, Bukharin et al. [42] reported that the genome of *Bacillus paranthracis* ICIS-279 is 5,247,312 bp long, comprising 5,210 genes with a GC content of 35.5% [42]. The research by de Sousa [43] found the genome of another *Bacillus paranthracis* strain to be 5,300,124 bp with 5,320 genes and a GC content of 35.4% [43]. Additionally, the studies by Diale [44] and Diale et al. [45] on *Bacillus paranthracis* strain MHSD3 reported a genome length of 5,284,112 bp, containing 5,420 genes and a GC content of 35.3% [44, 45]. Kumari et al. [37] analyzed a strain involved in a waterborne outbreak in Shandong province, China, with a genome length of 5,310,521 bp, 5,450 genes, and a GC content of 35.4% [46].

These studies utilized various sequencing technologies and bioinformatics tools. Next-generation sequencing (NGS) platforms such as Illumina HiSeq and PacBio RS II were commonly employed to generate high-quality genome sequences. Assembly tools like SPAdes and Canu facilitated de novo genome assembly, with some studies using hybrid assembly approaches combining short-read and long-read data. For genome annotation, tools such as Prokka, RAST, and the NCBI Prokaryotic Genome Annotation Pipeline (PGAP) were widely used. Additional functional annotation and pathway analysis were performed using tools like InterProScan, Pfam, and KEGG. Comparative genomics analyses employed tools such as Mauve and ProgressiveMauve for multiple genome alignment, along with BLAST for identifying homologous genes [41–48].

**Fig. 4:**
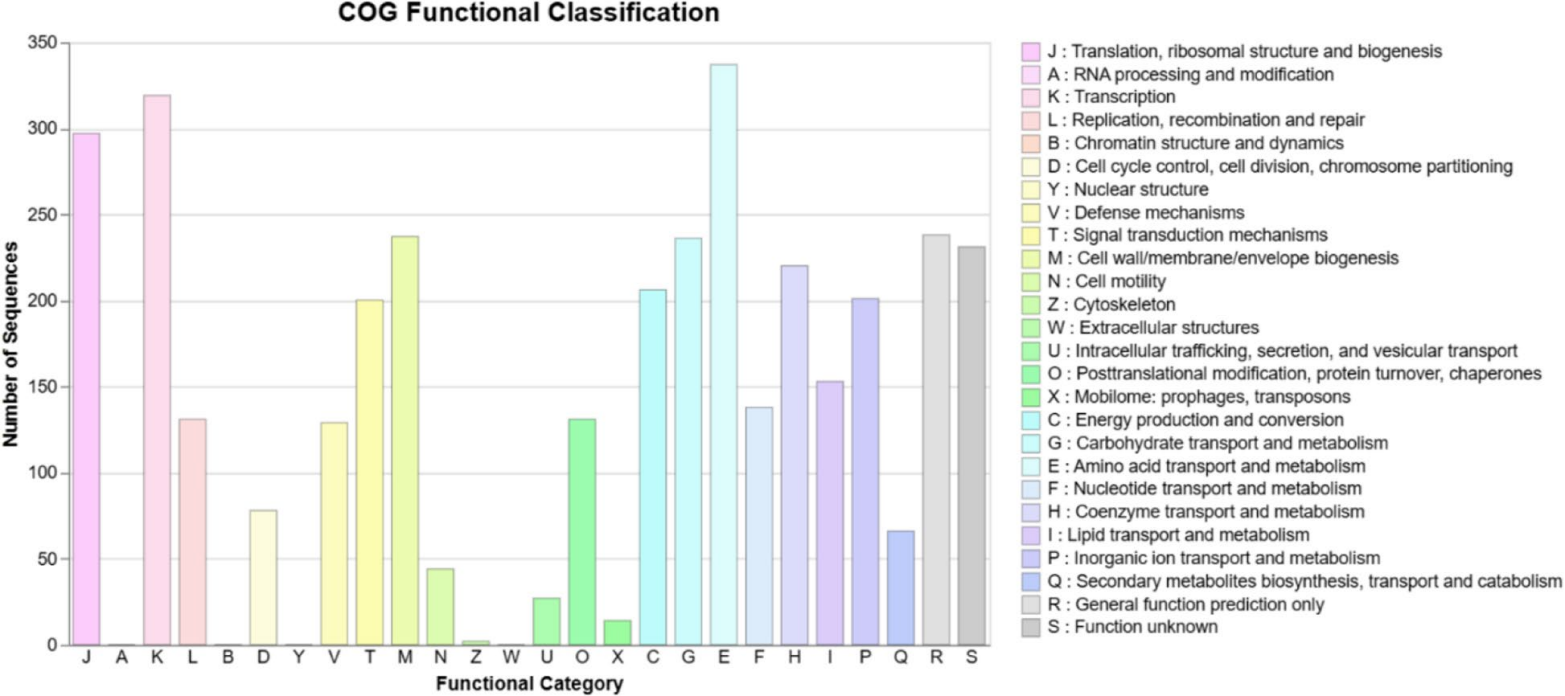
COG Functional classification of *Bacillus paranthracis* RSKS-3

The functional genome annotation of *Bacillus paranthracis* RSKS-3 covers various metabolic pathways and cellular processes (Table 11, Fig. 4). In the study, *Bacillus paranthracis* RSKS-3 had around 319 genes for amino acid metabolism and other amino acid metabolism. For carbohydrate metabolism, 266 genes were identified. For genetic information processing involving translation, transcription, folding; sorting; and degradation, and replication and repair, 81, 5, 40, and 65 genes were identified, respectively. For cofactor and vitamins metabolism, 151 genes were identified. For nucleotide metabolisms, 69 genes were identified. Similar studies were reported for amino acids and their derivatives, around 300 genes were identified, involved in the biosynthesis and metabolism of amino acids such as lysine, methionine, tryptophan, and histidine. Carbohydrate metabolism involved approximately 450 genes, including those encoding enzymes and transporters for glycolysis, gluconeogenesis, and the pentose phosphate pathway. Protein metabolism was represented by 380 genes encoding proteases, peptidases, and other enzymes. About 200 genes were involved in the biosynthesis of cofactors, vitamins, prosthetic groups, and pigments. Nucleosides and nucleotides metabolism included 220 genes involved in synthesis, salvage, and degradation processes. Regarding dormancy and sporulation, 150 genes were identified, including those encoding regulatory and structural proteins such as Spo0A and SpoII. Cell wall and capsule biosynthesis involved 180 genes, while RNA metabolism was represented by 130 genes encoding RNA polymerases and ribonucleases. DNA metabolism included 160 genes for replication, repair, and recombination, and fatty acids, lipids, and isoprenoids biosynthesis comprised 210 genes. Stress response genes numbered around 190, including those for heat shock proteins and oxidative stress response. Motility and chemotaxis involved 170 genes for flagellar biosynthesis and signaling pathways. Membrane transport was represented by 240 genes encoding various transporters and efflux pumps. Respiration processes included 160 genes for aerobic and anaerobic respiration. Virulence, disease, and defense mechanisms involved 230 genes related to toxin production, antibiotic resistance, and immune evasion. Lastly, phosphorus metabolism included 120 genes for phosphate acquisition, utilization, and regulation [41–48].

**Table 11:** Functional gene annotation of *Bacillus paranthracis* RSKS-3

LEVEL-1	LEVEL-2	GENE COUNT
METABOLISM	Carbohydrate metabolism	266
	Amino acid metabolism	242
	Metabolism of cofactors and vitamins	151
	Energy metabolism	129
	Nucleotide metabolism	69
	Lipid metabolism	51
	Glycan biosynthesis and metabolism	51
	Metabolism of other amino acids	53
	Metabolism of terpenoids and polyketides	31
	Biosynthesis of other secondary metabolites	37
	Xenobiotics biodegradation and metabolism	40
Genetic information processing	Translation	81
	Transcription	5
	Folding, sorting and degradation	40
	Replication and repair	65
Environmental information processing	Membrane transport	123
	Signal transduction	96
	Signal molecules and interaction	1
Cellular processes	Transport and catabolism	8
	Cell growth and death	14
	Cellular community—prokaryotes	73
	Cell motility	38

These comprehensive genomic analyses enhance our understanding of *Bacillus paranthracis* and its potential applications in various fields.

#### Phylogenetic analysis

**Fig. 5:**
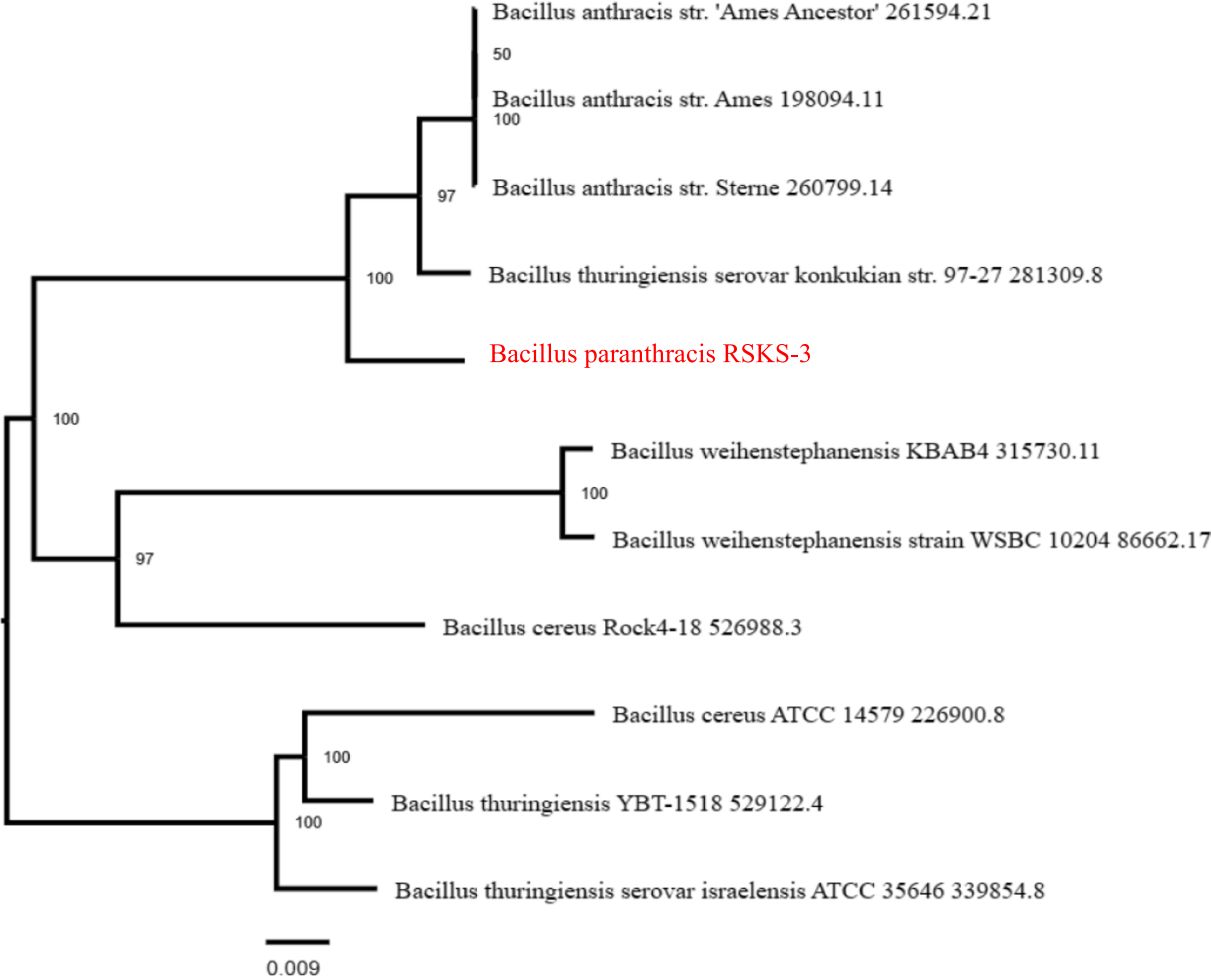
A *Bacillus* nucleotide sequence-based using a neighbor-joining phylogenetic tree was generated with Mega-X software displaying the position of *B*. *paranthracis* RSKS-3. 1000 replicates of book strap values are displayed at the nodes

Using the fastANI tool, 154 reference genomes of *B*. *paranthracis* were queried against the user strain (RSK-3) to determine the average nucleotide identity (ANI). Confirmation of the species level is based on values between 95 and 96%. Below are the top few lines of the fastANI findings. *B*. *paranthracis* strain 44.2 has the highest ANI value at 99.515, while *B*. *paranthracis* Bt C4, the representative genome, has the lowest ANI value at 97.7972. Genes with single copies were analyzed by the PATRIC server and used in RAxML to create a phylogenetic tree (Fig. 4). The reference and representative genomes are provided by PATRIC, and their contents are included in the phylogenetic analysis that Mash/MinHash generates. To determine the phylogenetic position of this genome, PATRIC global protein families (PGFams) were chosen from among these genomes. These families' protein sequences were aligned using MUSCLE, and each sequence's nucleotides were mapped to the corresponding protein alignment (Fig. 5).

#### PHA gene identification in *B*. *paranthracis* RSKS-3

**Fig. 6:**
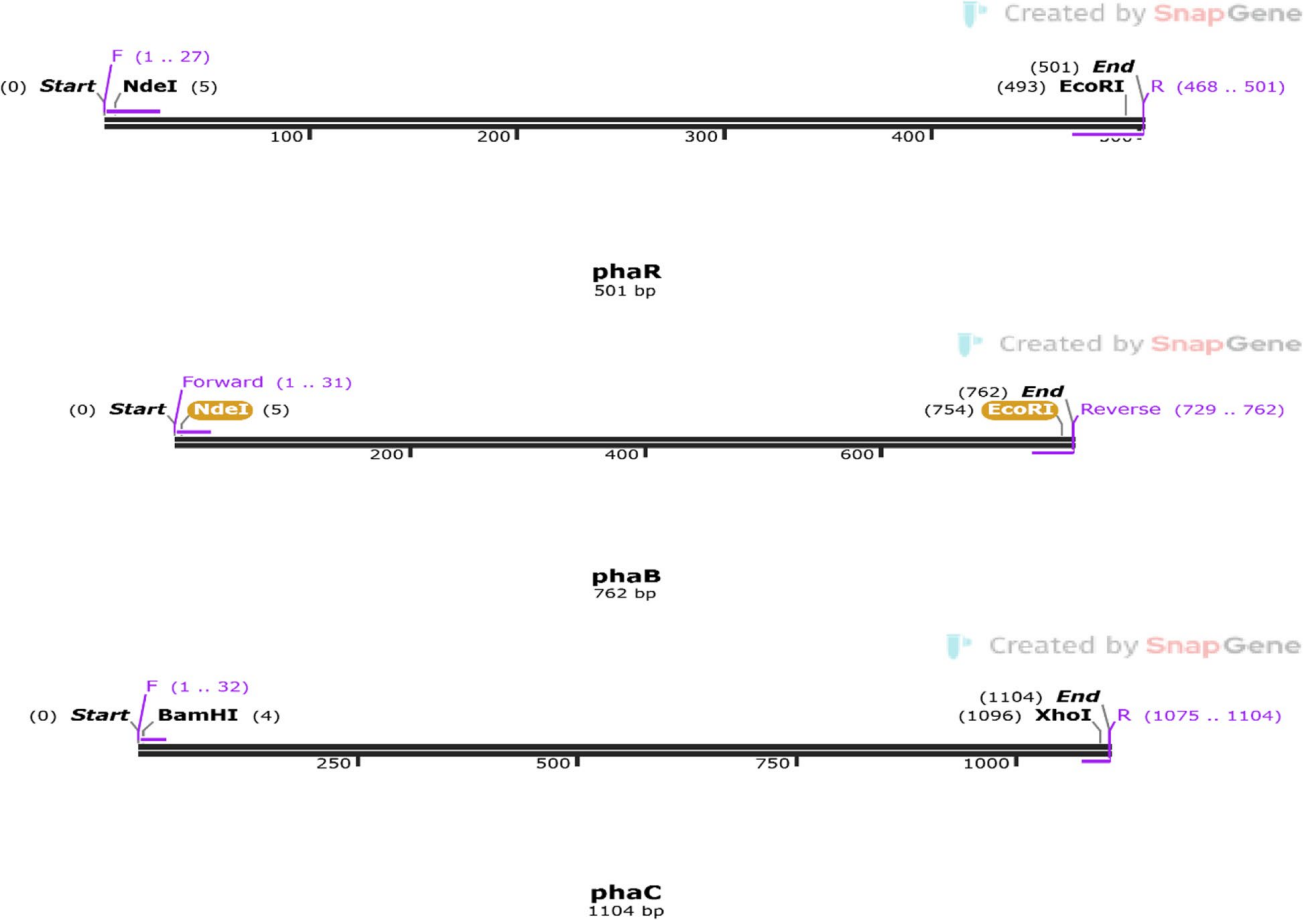
Fingerprint of restriction digestions of *pha* genes of *B*. *paranthracis* RSKS-3

The fingerprint analysis of restriction digestions of *pha* genes from *Bacillus paranthracis* RSKS-3 was conducted using SnapGene, a powerful molecular biology software (Fig. 6). The process involved the utilization of restriction enzymes to cleave the DNA at specific recognition sites within the *pha* genes, generating distinct fragments. The resulting restriction digestion patterns, akin to unique genetic fingerprints, were simulated and visualized in SnapGene to elucidate the structural variations within the genomic DNA. The simulated fingerprint of restriction digestions offered a predictive framework, guiding laboratory efforts in designing and interpreting actual restriction digest experiments.

The amplicons obtained from the PCR amplification were analyzed on a 1.5% agarose gel, and Fig. 6 shows the gel electrophoresis pattern that was established. The direct validation of effective gene amplification was provided by the appearance of bands on the agarose gel.

The amplified PCR products were compared with in-silico PCR simulations to further corroborate the experimental results. The experimental gel electrophoresis data were aligned with the theoretical PCR amplicons that were simulated using bioinformatics methods (Fig. 6). The congruence between the in-silico and actual results confirmed that the target genes (*phaR*, *phaB*, and *phaC*) were amplified accurately and successfully.

### Production and extraction of PHA

**Fig. 7:**
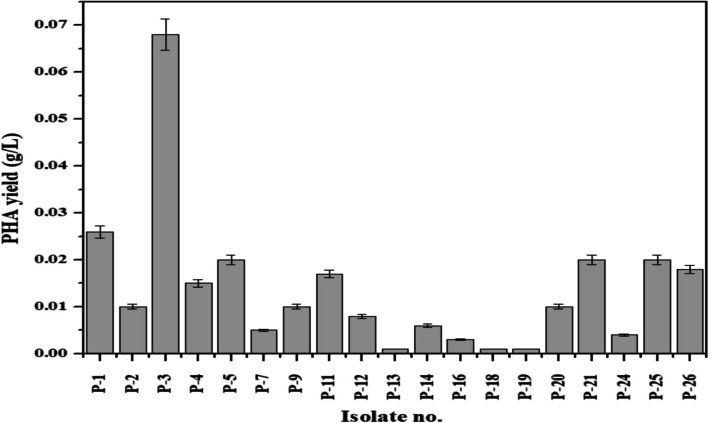
Polyhydroxyalkanoate production using different isolates

The investigation into PHA production among 26 microbial isolates (Fig. 7) yielded diverse outcomes, shedding light on the variability of biopolymer synthesis potential within microbial populations. Analysis of PHA yields revealed a range from 0.001 g/L to 0.068 g/L across the isolates, indicating significant differences in their capacity for bioplastic production. Notably, isolate P-3 demonstrated the highest PHA yield at 0.068 g/L, while P-9 exhibited the lowest yield at 0.001 g/L. These findings underscore the intrinsic variability in PHA production among microbial strains and the importance of screening multiple isolates to identify high-performing candidates.

Further examination identified isolates such as P-21 and P-25, alongside P-3, as particularly promising in terms of PHA productivity, with yields of 0.02 g/L and 0.02 g/L, respectively. Conversely, isolates like P-7 and P-13 displayed minimal PHA production, with yields below 0.005 g/L. This variability in PHA yields among the isolates suggests differential regulation of biosynthetic pathways and responses to environmental stimuli, highlighting the complexity of microbial PHA metabolism.

The initial findings reveal a PHA production of 0.068 g/L, markedly inferior to previously documented PHA-producing bacteria. The potential for PHA production differs among various bacterial strains and optimization methods. Oyewole et al. [49] indicated that *Pseudomonas aeruginosa* (OL405443) generated 54% PHA of CDW utilizing carbon sources supplied from agrowaste. [49]. Conversely, Mahajan et al. [50] attained a superior yield of 62% PHA from CDW in *Mesobacillus aurentius* via statistical optimization, illustrating the influence of process enhancement on polymer accumulation [50]. Moreover, Lee et al. [51] improved *Cupriavidus necator*'s capacity to metabolize xylose, yielding 70% PHA of CDW from a mixture of sugars. The reduced yield noted in *B*. *paranthracis* RSKS-3 may result from suboptimal growing circumstances, substrate deficiencies, or inherent metabolic restrictions [51].

This study is a preliminary examination; so, additional tuning is necessary to improve PHA accumulation in *B*. *paranthracis* RSKS-3. Our prior investigations have concentrated on optimizing fermentation conditions, selecting carbon sources, and addressing nutritional restrictions to enhance production [33–35]. Future studies may investigate metabolic engineering, co-cultivation techniques, or alternate feedstocks to optimize PHA production efficiency. Furthermore, comparative genomic study may elucidate its PHA biosynthesis routes, potentially facilitating genetic alterations to improve polymer synthesis. Although its initial yield is lower, the identification of *B*. *paranthracis* RSKS-3 as a PHA producer enhances the diversity of biopolymer-producing microorganisms, underscoring its potential for future industrial applications.

### PHA characterization

#### FTIR

An effective analytical method for examining the molecular structure and chemical makeup of polyhydroxyalkanoates (PHAs) is Fourier Transform Infrared Spectroscopy (FTIR). PHAs' FTIR spectra show discrete peaks linked to various functional groups, making it possible to identify key components such as aliphatic chains (C-H stretching) and ester groups (C = O stretching). This technique makes it easier to evaluate the PHA composition both qualitatively and quantitatively by supplying a way to calculate the relative amounts of different constituents in copolymers or blends. Furthermore, Polyhydroxyalkanoates (PHAs) can be examined for crystallinity using Fourier Transform Infrared Spectroscopy (FTIR), since changes in peak intensity and shape can provide information about the level of crystallinity.

**Fig. 8:**
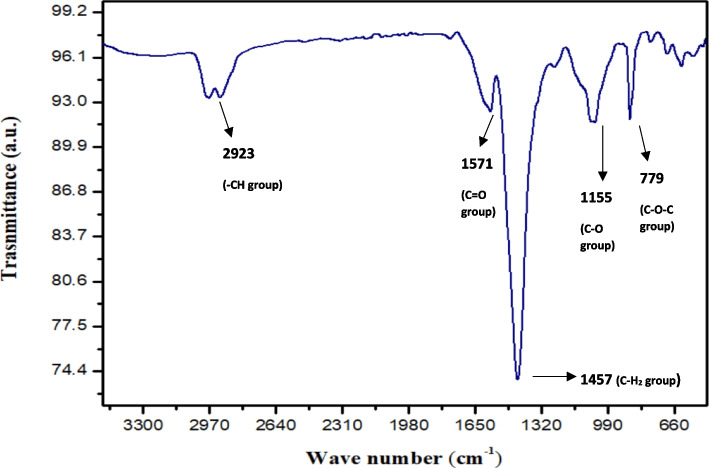
FT-IR analysis of PHA obtained from *B. paranthracis* RSKS-3. The 2923 cm^-1^ band corresponds to aliphatic -CH stretching, indicating -CH_2_- and -CH_3_ groups. The 1571 cm^-1^ and 1155 cm^-1^ bands represent C = O stretching, essential for ester linkage formation in the PHA backbone. The 1457 cm^-1^ band corresponds to CH_2_ bending, further confirming methylene groups. The 779 cm^-1^ band represents C–O–C stretching, characteristic of the ester group in PHA

The FTIR of extracted PHA is shown in Fig. 8. The absorption band at 2923 cm^−1^ corresponds to the stretching vibration of aliphatic -CH groups in the PHA backbone. This band indicates the presence of methylene (-CH_2_-) and methyl (-CH_3_) groups in the PHA structure. The absorption bands at 1571 cm^−1^ and 1155 cm^−1^ are attributed to the stretching vibration of the carbonyl (C = O) group in the PHA backbone. The carbonyl group is a key functional group in PHA, as it is responsible for the formation of the ester linkage between the hydroxyalkanoate monomers, which can influence the properties of PHA. The absorption band at 1457 cm^−1^ corresponds to the bending vibration of the aliphatic CH_2_ group in the PHA backbone. This band provides further evidence of the presence of methylene groups in the PHA structure. The absorption band at 779 cm^−1^ corresponds to the stretching vibration of the C–O–C bond in the ester group of the PHA backbone. This band confirms the presence of ester linkages between the hydroxyalkanoate monomers, which are characteristic of PHA polymers. These peaks coincide with the reported studies on polyhydroxyalkanoate production [52–54].

#### XRD

**Fig. 9:**
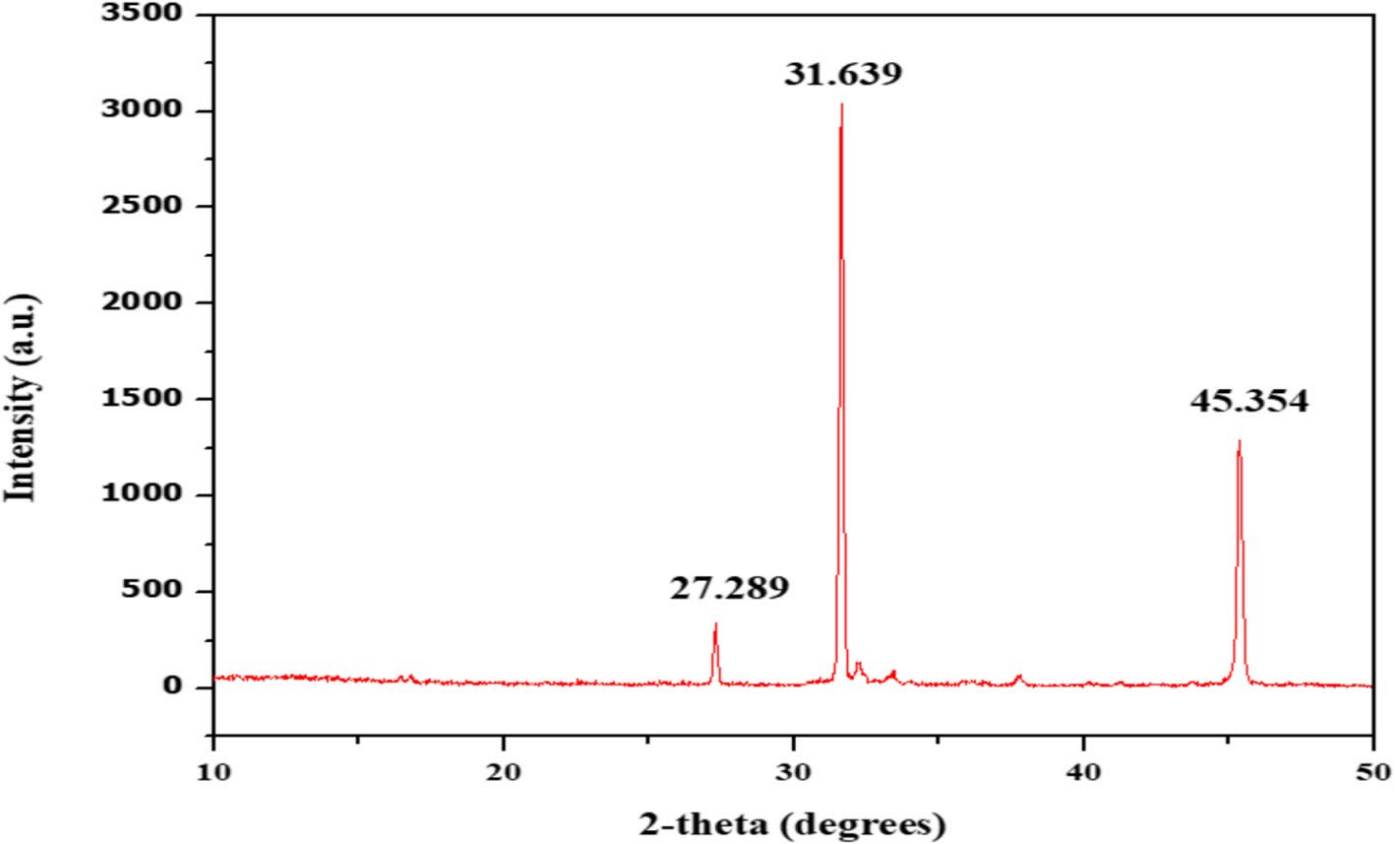
XRD graph representing peaks corresponding to PHA extracted from *B*. *paranthracis* RSKS-3. The peaks at 27.289°, 31.639°, and 45.354°**,** indicating its semi-crystalline nature and ordered molecular structure

An additional insightful method for probing into the crystalline structure of polyhydroxyalkanoates (PHAs) is X-ray diffraction (XRD), a valuable analytical technique. By subjecting a sample to X-rays and scrutinizing the ensuing diffraction pattern, X-ray diffraction (XRD) studies can unveil details regarding the atomic structure of the material. Particularly in the realm of PHAs, XRD serves as a potent tool for assessing crystallinity degree, crystalline phases, and crystal orientation within the polymer. This analytical approach is particularly useful in PHA analysis, providing a means to determine the extent of crystallinity. Researchers can leverage the sample's diffraction pattern to differentiate between crystalline and amorphous regions within the polymer, enabling an in-depth analysis of the spatial organization and arrangement of crystalline structures in the PHA matrix. This analytical prowess is especially beneficial when navigating the intricacies of polyhydroxyalkanoate characterization. Expanding upon the exploration of polyhydroxyalkanoates (PHAs), X-ray diffractometry has been employed to assess their degrees of crystallization. Specifically, the correlation of PHA with theta values at 27.289, 31.639, and 45.354 has been investigated **(**Fig. 9**)**. The peaks at 27.289°, 31.639°, and 45.354° signify the semi-crystalline characteristics of PHA. The diffraction patterns indicate the distinct crystalline areas of polyhydroxyalkanoates, implying an organized molecular arrangement within the polymer structure. The intensity and sharpness of these peaks indicate the level of crystallinity, which affects the mechanical strength, thermal stability, and biodegradability of PHA [55, 56].

Notably, the intensity observed at 31.639 surpasses that of the other values, indicating the crystalline nature of PHA. The peaks align to reported research by Rao et al. [57] and Grigary et al. [57, 58].

#### SEM

The examination of polyhydroxyalkanoates (PHAs) through scanning electron microscopy (SEM) proves instrumental in garnering valuable insights into the surface morphology and structure of these environmentally friendly polymers. This analytical technique facilitates a comprehensive exploration of the material's surface at both micro and nanoscales, revealing features such as granules, surface roughness, and overall texture. Additionally, SEM allows for the precise quantification of particle size and dispersion, providing essential information on the uniformity of polyhydroxyalkanoates (PHAs). The captured images highlight specific surface characteristics, including pores or fissures, which significantly influence the material's properties.

The application of SEM extends to examining the interaction between PHAs and other substances, especially in composite materials or blends. Moreover, it proves invaluable in degradation studies, enabling the analysis of changes in surface morphology over time by comparing images taken before and after degradation. In the context of PHA manufacturing, SEM plays a pivotal role in quality control, ensuring consistency and reliability in the production process. The microstructural investigations conducted by SEM contribute to a deeper understanding of PHAs, facilitating their optimization for diverse applications in sustainable and biodegradable materials.

**Fig. 10:**
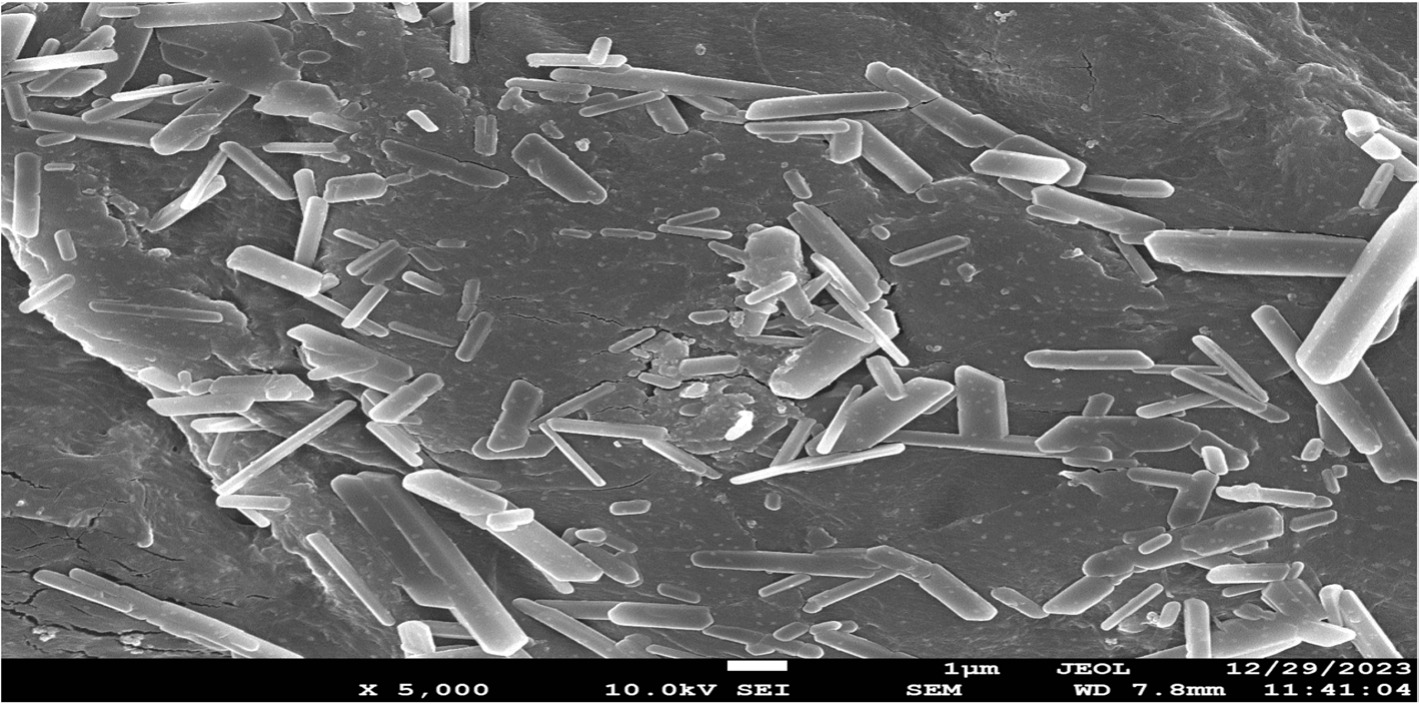
Scanning Electron Microscopy showing surface morphology of PHA of *B*. *paranthracis* RSKS-3

For instance, SEM analysis at a magnification of 2500X was employed to scrutinize the structure and shape of standard PHA (Fig. 10). The observed granules exhibited uneven shapes, existing either individually or in clusters. Furthermore, the extracted polyhydroxyalkanoate (PHA) from *B*. *paranthracis* RSKS-3 manifested in rectilinear forms of varying dimensions, aggregated together.

#### UV spectroscopy

**Fig. 11:**
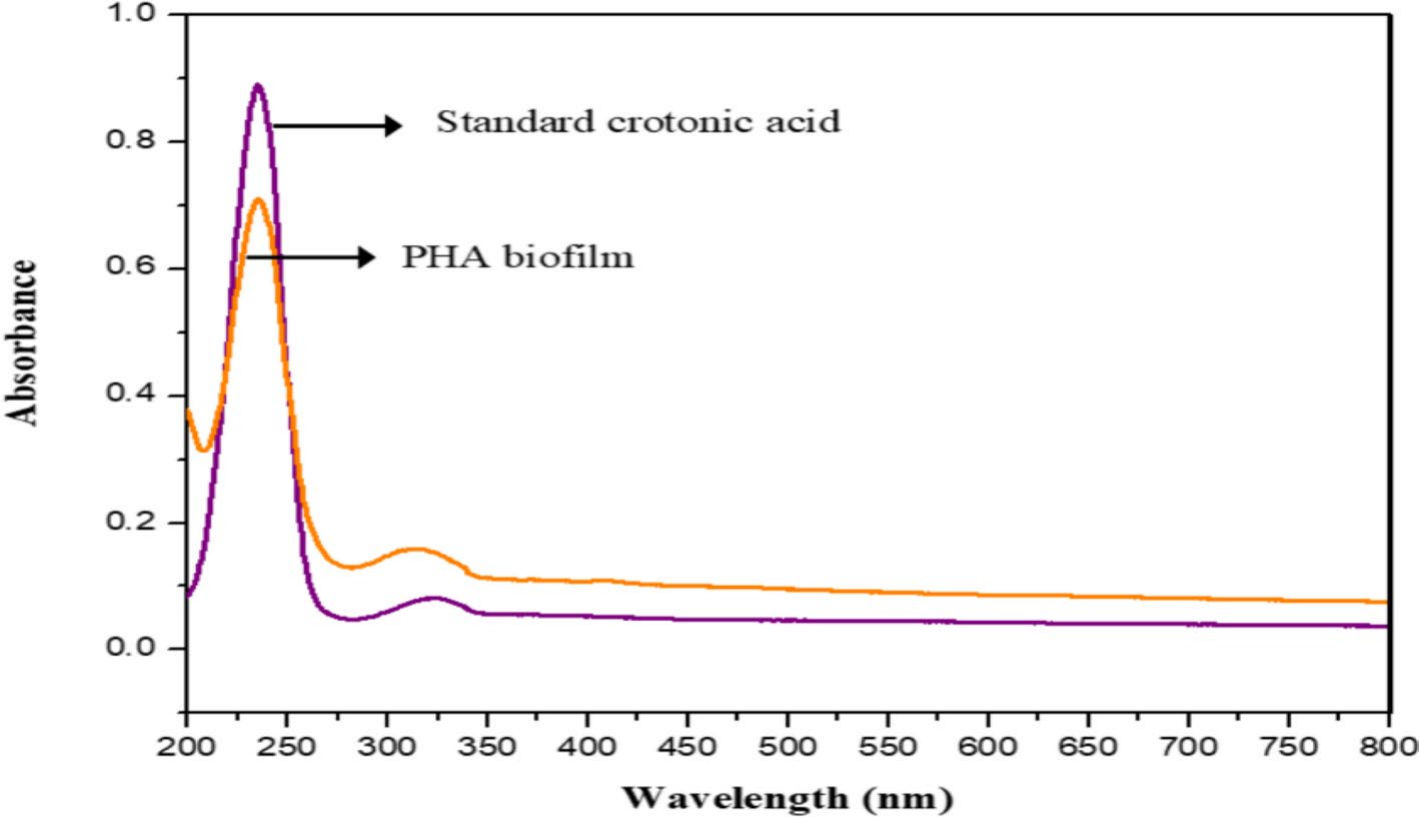
UV spectroscopy analysis of PHA (orange line) and crotonic acid (purple line)

The particular UV absorption peak at 235 nm indicates that, in the presence of concentrated sulfuric acid, polyhydroxyalkanoates (PHA) are converted to crotonic acid (Fig. 11). This peak acts as a recognizable signal that shows the sample contains PHA. Unquestionably, the development of crotonic acid—which is identified by a unique peak in the UV spectrum—provides evidence that the original polymer has undergone a chemical change. The unique UV signal at 235 nm, which identifies the converted product and correlates to the presence of crotonic acid, makes the confirmation crucial. Therefore, the combination of the observed change and the associated UV spectroscopic properties verifies that the substance that was subjected to concentrated sulfuric acid treatment consisted of polyhydroxyalkanoates [35].

#### NMR

Proton (H-NMR) and carbon-13 (C-NMR) investigations are two types of nuclear magnetic resonance (NMR) spectroscopy that are useful for deciphering the intricate molecular structures of polyhydroxyalkanoates (PHAs). H-NMR spectroscopy provides detailed information about the PHA monomer units, making it possible to pinpoint particular proton environments and make sense of the placement of hydrogen atoms along the polymer chain. To help determine the polymerization process, the spectrum also offers information on the protons at the ends of the polymer chains. Furthermore, PHAs have a variety of carbon homes, as shown by C-NMR spectroscopy, with peaks corresponding to different carbon types that provide important details about the polymer's composition. The chemical shifts seen in C-NMR spectra provide additional characterization of carbon atoms present in both the.

**Fig. 12:**
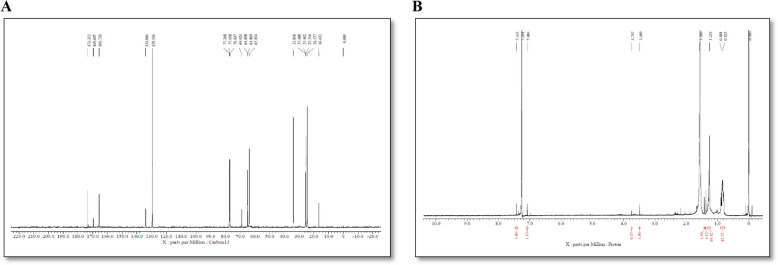
NMR analysis, where, (A) C’ NMR (B) H’ NMR of extracted PHA from *B*. *paranthracis* RSKS-3

Using 1H and 13C NMR spectra, the monomeric structure of the chemical extracted by *B*. *paranthracis* RSKS-3 was identified; the results are displayed in Fig. 12 (A and B). The solvent, CDCl_3_, was identified as the source of the signal at 1.2 ppm in the 1H NMR spectrum. A single-proton asymmetric carbon atom is next to the methylene (-CH–(CH_2_)–CO–) group, which is responsible for the peaks detected at 3.479 and 3.747 ppm. At 5.2 ppm, signals in the 1H NMR spectrum corresponding to methane (-CH-) protons were detected, suggesting the existence of -O-(CH-) CH_2_- linkage at carbon number 3. However, the signal in the 13C NMR spectrum seen in Fig. 11(B) between 16.651 and 33.856 suggests that the polymer contains methylene groups. The PHA polymer's side chain is shown by the peaks between 63.854 and 77.268. Finally, the signals between 165.730 and 173.313 show the existence of carbonyl groups attached to ester bonds, which are also present in the polymer. These peaks are consistent with already published reports [59–62].

#### TGA

A vital instrument for comprehending the thermal properties of polyhydroxyalkanoates (PHAs) is thermogravimetric analysis (TGA). TGA involves measuring the PHA sample's weight loss while exposing it to carefully calibrated temperature variations. Important new information on the heat stability and breakdown properties of PHAs is provided by this study. The first loss of mass, which is frequently linked to the release of volatile substances, signifies the start of degradation. The decomposition profile provided by the TGA curve sheds light on the many phases of the degradation process and the complex nature of thermal breakdown. Furthermore, TGA makes it easier to evaluate degradation byproducts and, when combined with techniques like gas chromatography-mass spectrometry (GC–MS), makes it possible to identify and quantify individual breakdown components. The evaluation of the thermal stability of different formulations of polyhydroxyalkanoate (PHA) is facilitated by comparative thermogravimetric analysis (TGA) studies, which in turn helps to improve the properties of polymers for a variety of applications. Additionally, TGA is essential to quality control in PHA production since it ensures consistency and predicts how the material will behave at various temperatures.

**Fig. 13:**
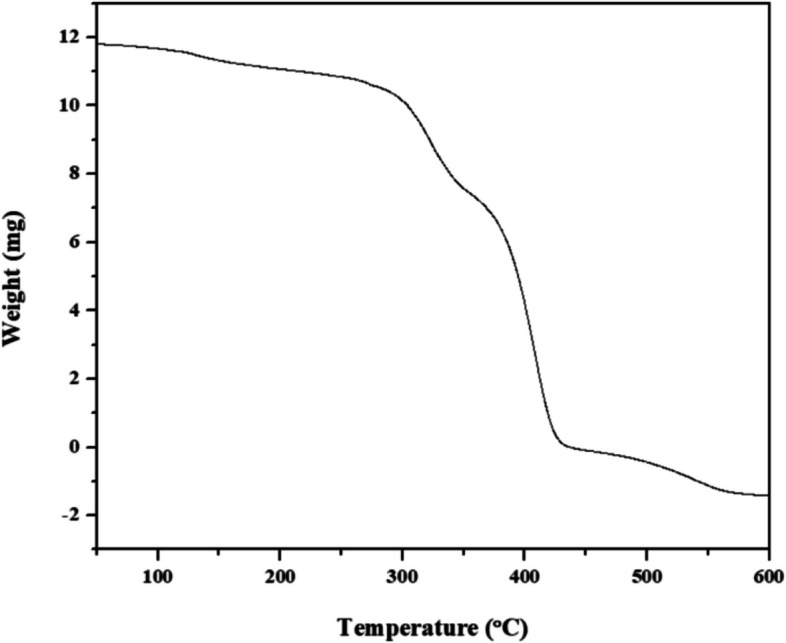
Thermo-Gravimetric Analysis of extracted PHA from *B*. *paranthracis* RSKS-3

Thermogravimetric analysis (TGA) was used to ascertain the PHA granules' thermal stability in the air. The TGA curve of the PHA isolated from *B*. *paranthracis* RSKS-3 up to 600 °C is shown in Fig. 13. The polymer was stable at 388.01 °C, and above this temperature, polymer degradation was commenced, according to the thermal degradation onset (T¬onset) data. Maximum sample weight loss (T¬max) was attained at 436.97 °C, and approximately 98.01% of the maximum weight loss was attained.

## Conclusion and future work

A major group of microorganisms isolated, screened, and characterized was gram-positive rod-shaped bacteria. A total of 26 potent PHA-producing bacteria were identified from sewage water in Punjab: Buddha Nala of Maheru village. Many of the bacteria isolated belonged to the genus *Bacillus* which dominated the group of PHA-accumulating bacteria. *B*. *paranthracis* RSKS-3 was reported to produce high-yield PHA during the study. Future work will focus on implementing Design Expert 12.0 to optimize and enhance *B*. *paranthracis* RSKS-3's potential to produce PHA. The findings of this study suggest a novel high-potential PHA production by bacteria isolated from the sewage water of Buddha Nala from Maheru, Punjab.

This exploratory work necessitates additional research to optimize *Bacillus paranthracis* RSKS-3 for improved PHA generation, especially by utilizing agrowaste as a cost-effective and sustainable substrate. This strategy may optimize yield and decrease production expenses, enhancing the feasibility of large-scale implementation. Nonetheless, increasing PHA production poses obstacles, such as preserving bacterial stability, optimizing fermentation parameters, and guaranteeing uniform polymer quality. The utilization of sewage water as a source of PHA-producing bacteria presents environmental problems, including unpredictability in microbial composition, potential contamination risks, and the existence of pathogenic species, necessitating comprehensive evaluation for biosafety and regulatory compliance. Notwithstanding these apprehensions, sewage water persists as an underutilized resource with considerable promise, providing a varied reservoir of industrially significant bacteria that may be harnessed for biopolymer synthesis and other biotechnological applications. Subsequent research should concentrate on process optimization, risk reduction, and the investigation of microbial diversity in sewage to uncover more effective PHA makers.

## Data Availability

The whole genome sequence of Bacillus paranthracis RSKS-3 has been registered in GenBank with bio-sample accession number SAMN39897631.

## Abbreviations

PHA: Polyhydroxyalkanoate
FTIR: Fourier Transformed Infra-Red
XRD: X-Ray Diffraction
SEM: Scanning Electron Microscopy
UV: Ultra-Violet
NMR: Nuclear Magnetic Resonance
TGA: Thermo-Gravimetric Analysis
H_2_S: Hydrogen Sulfide
DNA: Deoxy-Ribo Nucleic Acid
NCBI: National Center for Biotechnology Information
PGAP: Prokaryotic Genome Annotation Pipeline
KEGG: Kyoto Encyclopaedia of Genes and Genomes
KO: Kegg Orthology
PANTHER: Protein ANalysis THrough Evolutionary Relationships
GO: Gene Ontology
COG: Cluster of Orthologous Genes
GBDP: Genome BLAST Distance Phylogeny
GGDC: Chinese Genomics Data Center
PCR: Polymerase Chain Reaction
NaClO: Sodium hypochlorite
ATR-FTIR: Attenuated Total Reflectance Fourier Transform Infrared
EDS: Energy-dispersive X-ray spectroscopy
CDCl_3_: Deuterated chloroform
UPGMA: Unweighted Pair Group Method with Arithmetic Mean
ANI: Average Nucleotide Identity
PATRIC: Pathosystems Resource Integration Center
